# Individuals Matter: Dilemmas and Solutions in Conservation and Animal Welfare Practices in Zoos

**DOI:** 10.3390/ani12030398

**Published:** 2022-02-08

**Authors:** Anne Safiya Clay, Ingrid J. Visseren-Hamakers

**Affiliations:** 1 Department of Environmental Science and Public Policy, College of Science, George Mason University, Fairfax, VA 22030, USA; 2 Department of Geography, Planning and Environment, Institute for Management Research, Radboud University, Nijmegen 6525, The Netherlands; ingrid.visseren@ru.nl

**Keywords:** zoos, conservation, animal welfare, animal rights, captive breeding, compassionate conservation, species, individual, intrinsic value

## Abstract

**Simple Summary:**

Reaching conservation objectives while upholding individual animal welfare standards presents a significant challenge for zoos, especially if some individual animal interests conflict with their conservation mission. However, the compassionate conservation movement offers a potential solution for facing such challenges by advocating for the consideration of individual animal interests as central to conservation decision making. Our objective is therefore to determine to what extent zoological parks recognize the intrinsic value of zoo animals, beyond being members of species or ecosystems, and how this recognition manifests. Through discourse analysis, interviews, and relevant printed sources, we analyze the discourses, or concepts and categorizations, by which actors—experts in the conservation, animal rights, welfare, and zoo fields—give meaning to zoo practices. We demonstrate ways in which these discourses shape the captivity, breeding, and culling practices of individual zoo animals in the name of conservation. We found that people justifying these practices within zoos fail to recognize the intrinsic value of individual animals beyond being members of species. However, within the zoo, welfare practices and education objectives increasingly focus on fulfilling individual animal interests.

**Abstract:**

Compassionate conservation advocates for minimizing individual suffering in conservation practice and adheres to the principle “individuals matter”—intrinsically, in and of themselves. Our objective is to determine the extent to which, and how, zoos recognize the intrinsic value of wild individuals beyond their status as members of species or ecosystems. We analyzed discourses surrounding the Smithsonian National Zoo in the U.S.A., the zoos of the Muséum National d’Histoire Naturelle in France, and the Seoul Grand Park Zoo in South Korea. Using existing literature on zoos, conservation, animal welfare, and rights, we distilled two discourses (justificatory and abolitionist). Through interviews with professionals in the zoo, conservation, welfare, and animal rights communities, we demonstrate how actors frame individual zoo animals as (1) sentient persons, (2) reproductive components, and (3) species ambassadors. Our analysis shows how actors’ views shape three zoo practices related to ex situ conservation: (1) captivity, (2) captive breeding, and (3) culling. This analysis revealed two significant findings. First, actors representing the justificatory discourse fail to frame animals as intrinsically valuable individuals. Second, within the constraints of the zoo, the intrinsic value of individual animals is recognized through welfare practices and education focused on fulfilling animal interests.

## 1. Introduction

Compassionate conservation maintains that, since both conservation and animal welfare ethics care about nature, both should work together to maximize conservation outcomes and minimize harm to individual, non-human animals (hereafter referred to as animals). This field of conservation attempts to combine two ethical perspectives that conventional conservationists have deemed “conceptually distinct” and “politically separate” [1] (p. 731). Though interdisciplinary and morally pluralistic, compassionate conservation binds its proponents by a set of foundational principles that advocate against intentional harmful, lethal, and invasive actions towards individual animals [2]. Reflecting Leopold’s (1949) holistic view of nature, where “a thing is right when it tends to preserve the integrity, stability, and beauty of the biotic community”, and “wrong when it tends to otherwise”, traditional conservation practice has historically favored the preservation of biodiversity through the protection of biotic wholes, their evolution, and genetic diversity [3] (p. 217). Soulé (1985) defines conservation as a value-driven and value-laden science through normative postulates, stating that biotic diversity has intrinsic value, and that ecological complexity and evolution are good. A traditional conservation ethic therefore tends to place the moral value of species and ecosystems above their individual components, permitting conservation management methods that may be detrimental to individual animals but beneficial to the diversity, complexity, and evolution of the populations and landscapes they comprise [1]. Developed out of concern for individual animals, the compassionate conservation movement challenges such practices by placing the well-being of animals at the center of conservation decision making.

A key premise of compassionate conservation is that individuals matter [4,5,6]. This means that one must “acknowledge the intrinsic value of wildlife and resist the tendency to reduce them or their value solely to their position as members of collectives” [6] (p. 1260). Stating that individuals matter requires a recognition of their intrinsic value, beyond the instrumental value associated with maintaining the genetic diversity and complexity of collective entities. At the same time, recognizing the intrinsic value of individual animals also signifies an understanding that their well-being is deeply entangled with the health of their communities. Because “individuals are repositories of social and practical knowledge for their groups and they provide social and behavioral stability”, they hold as much conservation value as the collectives they are a part of [7] (p. 1). Conventional conservation does warn against underestimating the importance of “conducting scientific and technological studies of individual components” to “grasp the functional intricacies of complex systems” [1] (p. 728). However, its practices have historically prioritized genetic and evolutionary processes to maintain the existence of species and populations. Concern for the welfare of individual animals has been considered neither “necessary [nor] desirable for conservation biology” [1] (p. 731). In contrast, though compassionate conservation does not deny the importance of collectives in conservation work, its proponents express concern that “a singular focus on the protection of wildlife collectives is ethically indefensible to the extent that it blinds conservationists to the wrongs enacted against wildlife individuals” [6] (p. 1262).

Conservation and animal welfare both represent distinct but partly overlapping ethical perspectives; yet, they also diverge dramatically [8,9,10]. Both perspectives, for example, condemn practices such as illegal poaching, but may disagree when it comes to the moral acceptability of culling an invasive animal. Where conservation works to maintain the biotic integrity of populations, species, and/or ecosystems, welfare prioritizes the quality of life of individual sentient beings and works to prevent their suffering at the hands of humans. Although there is no set universal definition for animal welfare, the Five Domains model represents the most current, evidence-based framework, used to recognize and classify positive and negative welfare states and their interactions in different settings [11]. An update on the “Five Freedom’s model“, based on advances in animal welfare science, it includes the following domains: (1) Nutrition, (2) Physical Environment, (3) Health, (4) Behavioral Interactions, and (5) Mental State; moreover, it offers captive animal facilities a grading system to determine, among other factors, how the first physical domains positively or negatively affect the fifth mental state domain, which represents an individual animal’s affective experiences [12,13]. As an ethic, however, animal welfare allows certain harms to individuals, such as euthanasia or confinement, as long as the individual suffers minimally and the resulting benefits to other individuals or collectives outweigh the cost of that suffering [4]. This differs from the animal rights perspective, which takes the welfare concept further by attributing non-human animals with personhood and intrinsic rights to life, liberty, and bodily integrity [14]. However, both views prioritize the moral considerability of individual animals [5,15].

The concept “modern zoos of the twenty-first century” refers to organizations accredited by regional or world zoo associations. Such zoos are held up by the five pillars of education, conservation, research, animal welfare, and entertainment [16]. The World Association of Zoos and Aquariums (WAZA) maintains that the “core purpose” of modern zoos is to practice conservation and that they must also uphold their “core priority” towards animal welfare [17]. In other words, zoo actions for conservation must also adhere to animal welfare standards that ensure individual animals have a life worth living [11]. WAZA facilitates collaborations and exchanges between institutional members of regional zoo associations, such as the European Association of Zoos and Aquariums (EAZA) and the Association of Zoos and Aquariums (AZA). All these associations set conservation objectives and ethical standards for their members. The adoption of the term “modern zoo” reflects the attempt of such zoological parks to distance themselves from the historical baggage of princely menageries, cabinets of curiosity, and amusement park-like “roadside zoos”, that show little regard for environmental education or animal interests. Many proponents of modern zoos advocate to drop the word “zoo” altogether, in order to redefine these institutions as twenty-first century conservation centers that care for animals using the best welfare practices available [18,19,20,21]. However, the official narrative of the modern zoo also risks making invisible the controversial ethics that surround life in captivity. By focusing this narrative on current efforts to save collective species, zoos tend to center on what animals represent, erasing the captive individuals themselves [22].

Achieving conservation objectives while upholding welfare standards presents a significant challenge to modern zoos that claim to have joined the frontlines in the fight against extinction. This is especially true if individual animal interests conflict with conservation priorities. In zoos, individual animals have come to represent their counterparts in the wild. Within this framework, zoos present education, conservation, and even animal welfare as valid justifications for captivity, as well as for invasive and sometimes lethal procedures towards individual animals [23,24]. However, the compassionate conservation movement advocates for the consideration of individual animal interests and, in some cases, recognizes the existence of personhood in all sentient beings in efforts to maximize conservation outcomes [25]. Although the current compassionate conservation movement is primarily anti-zoo, arguing that in situ conservation represents a more efficient and superior moral alternative, the compassionate conservation approach could potentially help address the tradeoffs that zoos face when juggling conservation and animal welfare priorities [4,26,27]. As institutions that have evolved under societal scrutiny, zoos have found different ways to respond to rising anti-zoo sentiments that deem that the captivity of wild animals is exploitative of their interests, well-being, and fundamental rights. Zoo professionals and animal rights and welfare advocates are engaged in fierce debates on what constitutes individual animal interests and to what extent these should be considered when saving species. These debates consist of several evolving discourses both within and outside of zoos, that influence the mindsets of zoo professionals, who seek solutions to ethical predicaments through the development of more positive animal welfare best practices.

This project looks at public zoos in the United States, France, and South Korea, all of which are members of their respective regional associations and WAZA. The objective of this paper is to determine the extent to which, and how, zoological parks recognize the intrinsic value of wild individuals, beyond their status as members of collective species or ecosystems. Using existing literature on the history and evolution of zoos, conservation, animal welfare, and animal rights, we distilled two fundamental discourses: the justificatory discourse of, among other domains, zoos, and the abolitionist discourse of animal rights proponents. Through document analysis and interviews with actors in the zoo, environmental, academic, and animal rights communities, we demonstrate how actors representing these discourses frame three distinct perceptions of individual zoo animals within modern zoos: first, as sentient persons, second, as reproductive components, and third, as species ambassadors. Our analysis focuses on three zoo practices as case studies: (1) captivity, (2) captive breeding, and (3) culling. We selected these three because they are restrictive or invasive to individual animals and represent ex situ conservation practices that zoos claim benefit collective species and ecosystems.

This article is structured as follows. We first begin with an overview of our theoretical framework, which represents a discourse analysis approach using discourse as frame. In the next section, we present our methodology. In Section 4 that follows, we introduce the discourses and organize our analysis according to three case studies consisting of zoo practices related to ex situ conservation. We demonstrate how actors representing each discourse frame individual animals, and how they situate the meanings of conservation and animal welfare to justify or condemn each practice. Next, we interpret and summarize our findings in the conclusion and discussion.

## 2. Theoretical Framework

We apply discourse theory, taking a discourse analysis approach, using discourse as an analytical tool in combination with frame theory to compare how actors within and outside zoos address dilemmas in practices that disregard the intrinsic value of individuals in favor of collectives. Foucault coined discourse as a “system of representation”, where people are trapped within the dominant discourses of the period in which they live [28] (p. 72). Interested in the rules and practices that regulate discourse in history, he argued that discourse both constructs and defines knowledge through language. He also maintained that all practices are discursive and that discourses are “practices that systematically form the objects of which they speak” [29] (p. 55). Approaches to analyzing discourse can range from “thick” to “thin”. Foucault’s discourse analysis represents a “thick” theoretical approach, where all reality is discursive and socially constructed. In contrast, the “thin” approach differentiates between the discursive and non-discursive, with discourses being one factor among many others to explain social phenomena [30]. Our discourse analysis, using discourse as frame, falls under the “thick” approach, and does not distinguish discourses from practices.

We define discourse as “an ensemble of ideas, concepts, and categorizations that are produced, reproduced, and transformed in a particular set of practices and through which meaning is given to physical and social realities” [31] (p. 67). Discourses are important because they “shape the perspectives of actors, while the latter, in turn, can reshape the former” [30] (p. 58). This aspect of discourse, known as reflexivity, describes the relationship between language and reality, where language both reflects and constructs the situation or context in which it is used [32]. Because discourses construct and are constructed by giving and acquiring meaning from reality, they become part of a system of repetition, which causes them to remain stable until actors reframe these discourses in the media, science, and politics [33].

Nestled within this system of repetition is the way actors frame their reality. We combine discourse theory with frame analysis to determine how actors frame problems or situations within their own minds and within the social networks they comprise. According to Hajer (1995), “Discourse analysis investigates […] how a particular framing of an issue makes certain elements appear fixed or appropriate while other elements appear problematic” [34] (p. 54). Specifically, Schön and Rein (1994) define frames as “the broadly shared beliefs, values and perspectives familiar to the members of a societal culture and likely to endure in that culture over long periods of time, on which individuals and institutions draw in order to give meaning, sense, and normative direction to their thinking and action in policy matters” [35] (p. xiii). Additionally, Perri (2005) underlines that frames function both to “organize experience” and create “bias for action” [36] (p. 94). In other words, frames represent clusters of knowledge that exist within discourses and are used by actors to make sense of their respective world views. Somorin et al. (2012) argue that frames are both influenced by and exist within overarching discourses [37]. As a result, determining whether zoos truly recognize the intrinsic value of wild individuals requires us to understand how actors frame the roles of individual zoo animals within the discourses they, respectively, represent. Understanding these frames allows us to shed light on how wider discourses both create and solve dilemmas in zoo practices.

Finally, to identify frames and the discourses they are part of, we distill situated meaning—”an image or pattern that we assemble ‘on the spot’ as we communicate in a given context”—of key words or phrases in language to help identify broader frames and the discourses they are a part of [32]. In this way, we use situated meaning as a tool to inform the broader background of a situation where meaning is both constructed and constructive.

## 3. Materials and Methods

Methods included interviews, document analysis, and non-participatory observation. Through a review of literature on animal welfare, ethics, conservation, and the evolution of modern zoos, we distilled and renamed two main discourses—the justificatory discourse of zoos, and the abolitionist discourse of animal rights—on the role of individual animals in zoological parks. The literature review provided a broader context in which to place the situated meaning of “conservation”, “welfare”, and “animal rights”, identified in the texts of in-person interviews conducted with actors in the conservation, animal welfare science, animal rights, and zoo communities. Although there are many thoughtful ethical and scientific critiques of zoo practices among a wide variety of intellectual perspectives and approaches such as virtue ethics, ecofeminism, and consequentialism, our interlocutors tended to draw their arguments from these two discourses related to the pro-zoo/anti-zoo debate on captivity and animal rights. We transcribed and subsequently analyzed each interview by distinguishing, coding, and categorizing patterns in their textual content. To guide us through the analysis, we used Gee’s (1999) seven questions for discourse analysis, which focus on the significance, practices, identities, relationships, politics, connections, and knowledge that the interviewees discussed [32]. We organized different passages of each interview transcription according to each of these seven categories and examined the contexts of words or passages that reflected situated meanings actors ascribed to “conservation”, “animal welfare”, and “animal rights”. These situated meanings allowed us to determine how actors framed individual zoo animals.

Interviewees were selected through the snowball approach, where research participants recruited or recommended other participants for the research. Each interview was free-form and semi-structured. The order of the questions varied, and follow-up questions ensued for clarification, depending on the flow of the interview. The length of the interviews also varied depending on the interviewee’s answers. On average, interviews were one hour in length, with the shortest being forty minutes, and the longest lasting up to three hours. Most interviews were conducted in person, with some interviews conducted remotely by telephone, Skype, or Zoom. Each interview proceeded either in English, French, or Korean, depending on the location and the interviewee’s language preference. Interviews were audio recorded, transcribed, and, when necessary, we used our fluent proficiency in each language to translate relevant citations into English.

Qualitative research requires a repeated process of data collection and analysis that must be continuously compared, contrasted, and verified [38]. To assure validity of the data, we maintained records in daily field journals, which included our initial thoughts surrounding each encounter before transcribing the audio recordings. These journal entries were then compared with our transcriptions of the audio files, and we recorded our follow-up impressions. Based on what initial interviews revealed, we also continuously refined or adjusted questions for future interviews with other research participants.

We received approval from George Mason University’s Institutional Review Board prior to conducting research in each field site. Most participants agreed to have themselves identified by name in this publication. Those who wished to remain anonymous will be described as representatives of their respective organizations.

Fieldwork in the form of non-participatory observation was conducted in each geographical site for several months at a time. In France, the research was hosted by the Centre d’Écologie et des Sciences de la Conservation (CESCO) lab at the Muséum National d’Histoire Naturelle (MNHN) for 15 months. In South Korea, field research was hosted by the EcoScience lab at Ewha Women’s University for 12 months. Finally, in the United States, field research was hosted by George Mason University’s (GMU) department of Environmental Science and Policy. GMU is affiliated with the Smithsonian Conservation Biology Institute (SCBI). In each site, we collaborated with local researchers in natural and social scientific research, participated in symposiums on zoo animal welfare, conservation, and reintroductions, connected with animal rights and welfare organizations, and visited local conservation organizations and facilities.

Situating this study in three different countries demonstrated how the discourses and dilemmas concerning animal welfare and conservation in zoos flow beyond cultural barriers. This also sheds light on what practices actors deem problematic and what solutions they apply in response to certain issues. Even though each site represents a distinct cultural context, they share many features with each other, as modern zoological parks seeking to balance a set of organizational and public priorities.

In France, we focused on MNHN’s three zoological parks: the Ménagerie of the Jardin des Plantes (hereafter referred to as the Ménagerie), the Paris Zoological Park (PZP), and the Haute Touche Zoological Reserve (RZHT). As one of the first public zoos in the world, the Ménagerie rose from the ashes of the royal menagerie of Versailles, with the idea that the nation would benefit from having live animal collections available for scientific instruction. Founded in 1792, this national zoo embodied the values of the French Revolution, which emphasized equality, education, and rationality [39]. Presently, it contains 600 individuals of 150 different species and covers 6 hectares. Exhibits focus on small–medium-sized species, in an effort to provide adequate space within historically classified enclosures, which house animals in early nineteenth century “fabriques” (farmhouse cabins with thatched roofs) and mid-twentieth century, art-deco style buildings [40]. Meanwhile, the larger PZP spans 14.5 hectares and contains more than 2500 individuals of 245 species. Located in Paris near the Bois de Vincennes, it first opened in 1934 and was closed in 2008 for 6 years of intense renovations. In 2014, the zoo reopened with a completely new layout of 5 bio zones, displaying immersive multi-species exhibits: (1) Patagonia, (2) Africa, (3) Europe, (4) Madagascar, (5) Amazon–Guyana [41]. It is the first zoo in the world to have been completely reconstructed and aims to emblematize a “new species of zoo”, as “a modern place of conservation”, that also “puts forward animal welfare and allows you to observe and discover the wild behavior of animals” [42]. Finally, the RZHT is the largest of the 3 MNHN zoos, spanning 436 hectares and containing 1300 individuals of 120 different animal species. Located 282 km outside of Paris, its vast enclosures make it the most spacious zoo in France. Originally founded in 1958 as a breeding center for the Ménagerie and the PZP, the RZHT inaugurated its own research laboratory in 2000, which prioritizes reproductive research for conservation, such as in vitro fertilization methods and artificial insemination [43] (Table 1).

In the Republic of Korea, we focused on the Seoul Grand Park Zoo (SGPZ). Originally founded in 1909 by Japanese colonizers during the occupation of Korea, the Seoul Zoo was moved to the mountains of Makgyedong, Gwacheon in 1984 as part of a large amusement park. A testament to South Korea’s rapid economic growth, this large park was meant to be akin to a Korean Disneyland. Currently, the SGPZ extends over 58 hectares and contains 1810 individual animals representing 132 species. It is the only research-oriented zoo in South Korea [9] (Table 2).

In the United States of America, our research centered on the Smithsonian National Zoological Park (SNZP) in Washington D.C. and the Smithsonian Conservation Biology Institute (SCBI) in Front Royal, Virginia. Where the SNZP covers 66 hectares and contains 2700 animals of 390 species, the SCBI spans over 1295 hectares and contains 20 species of animals (Table 3).

## 4. Results

In this analysis, we identified two fundamentally opposing discourses (abolitionist and justificatory) on the relationship between conservation, animal welfare, and animal rights in zoos. The abolitionist discourse maintains that only individuals matter and that every sentient being is a person with intrinsic rights to life, liberty, and bodily integrity [14]. However, in zoos, the question of whether “individuals matter” is often dismissed by a justificatory discourse, which justifies invasive actions on individual animals as necessary for zoo conservation biology [22]. We determined that actors using these two discourses framed individual zoo animals in three distinct ways. Actors representing an abolitionist discourse framed animals as (1) sentient persons; whereas, actors representing a justificatory discourse framed them as (2) reproductive components and (3) species ambassadors. Since these frames serve to inform zoo practices, we organized our analysis around three of the most contentious practices related to ex situ conservation in zoos: (1) captivity, (2) captive breeding, and (3) culling. We chose these three because actors indicated that they inherently presented the most ethical dilemmas between individual animal interests and species conservation. To establish the extent to which, and how, zoo animals were perceived as individuals, we identified the situated meanings actors ascribed to “conservation”, “animal welfare”, and “animal rights” for each practice. Because these meanings shape and support each practice, understanding them allowed us to determine what practices actors viewed as inherently appropriate or problematic and how they framed individual animals as a result.

### 4.1. Captivity

Placing animals in captivity for conservation education represents a central part of the justificatory discourse of modern zoos. The Ménagerie portrays its animal collections as comprising a “living book of animal diversity”, where “one can learn about what threatens these animals and the actions necessary to guarantee them a livable space” [44] (p. 11). For zoo actors, captivity enables visitors to be in proximity with live animals. Jacques and Quertier (PZP) believe such experiences inspire deep connections to animals that may lead to conservation action: “It’s true, we have TV, we have internet, we have books, but nothing beats the direct visual contact with an animal in the flesh. Everyone knows what a giraffe is, but to see a live giraffe, that makes all the difference”. Kim, B. (SGPZ), who views the zoo as “a living site of education”, shares this sentiment: “Reading thousands of books is incomparable to looking one time into an animal’s eyes”.

To fulfill this educational mission, we found that zoo actors representing a justificatory discourse framed individual zoo animals as “ambassadors” of their free ranging counterparts and the natural ecosystems they inhabit. Servais (ULiège) emphasizes that, “Historically, the vocation of the zoo was to present specimens, animals that were in a sense there to represent a species, but they were not there for themselves”. Within zoos, individuals therefore represent larger collectives, before themselves. Kim, B. (SGPZ) portrays this as fundamental to the zoo’s conservation mission: “Zoos are places meant to hold many animals. Each one represents his or her whole species in the wild. I believe all of them, as ambassadors of their counterparts, are being sacrificed for conservation”.

In contrast, actors representing an abolitionist discourse framed zoo animals as “sentient persons” with an intrinsic right to liberty. Captivity therefore violates the moral rights of individual animals by denying them freedom and the ability to express the complete inventory of their natural capacities. As Sanvisens (PAZ) states: “For me, [orangutans in the zoo] are not even orangutans—they’re prisoners. They don’t express their natural behavior. They are shadows of themselves”. For these animal rights actors, captive animals cannot be effective ambassadors because they are exploited. As Gérôme (FBB) states: “Today we have technologies like television and holograms that allow us to see and learn about species […] We can’t just imprison sentient beings capable of suffering for educational goals while claiming it’s pleasurable to see them”.

Anti-zoo animal rights actors representing an abolitionist discourse situated “welfare” as unable to provide captive zoo animals with a life worth living. For these groups, animal welfare is insufficient to make up for the loss of liberty of an individual animal. Sanvisens (PAZ) explains, “The notion of animal welfare has always been put forward by enterprises or institutions that exploit animals. It is not a notion where we recognize ourselves. It’s not just about maintaining an animal alive—we want to go much further than that. For us, the notion of liberty is fundamental and notably for wild animals”. Morette (Code Animal) echoes this, expressing: “I abhor that concept. For me, it’s marketing. We prefer to speak of the animal condition. But animal welfare is a very subjective notion”. Parallel to this, Bachelard (LFDA) views animal welfare as applicable only to domestic animals dependent on human care. Defining welfare as the “positive mental and physical state as related to the fulfillment of [an animal’s] physiological and behavioral needs in addition to [his/her] expectations” [45]; Bachelard believes that, “technically, a captive animal in a zoo will never be able to reach a state of good welfare, [because] zoos are incapable of responding to their physiological and behavioral needs”.

However, South Korea’s main animal rights organization, Korean Animal Rights Advocates (KARA), stands together with welfare associations, such as Animal Welfare Awareness Research and Education (AWARE) and Action for Animals, taking both a pro-zoo and pro-welfare stance. Jeon (KARA) believes that, “Zoos should focus on native species, research, conservation, inspiring awe about animals, and education. They should have well-done exhibits that look like the wild”. KARA campaigns to “reset and not abolish” zoos, a position that does not completely align with Morette’s (Code Animal) mentality: “We don’t try to improve captivity for animals because we don’t consider this our role. We’re here to be an outside force and a more radical discourse that moves public opinion and political powers. Since we consider that captivity is not, in any case, suitable for animals, placing a bit of glitter to make them a bit better is not really our role”. According to Lee (AWARE) and Ma (Animal Happiness Lab), Korean animal rights groups—already few—rarely make the distinction between animal welfare and rights, advocating instead for the humane treatment of animals in captive settings. Jeon (Action for Animals) describes the basic principle of welfare as “identifying and eliminating risks inherent in using animals from birth until death”. This has been the focus of Korean animal rights campaigns in relation to zoos [9]. As a result, Korean animal rights advocates do not represent an abolitionist discourse and situate welfare as sufficient to provide individual zoo animals with a life worth living. Yet, the recently established organization Animal Liberation Wave demonstrates an increase in anti-zoo sentiments, calling for a boycott of “animal prisons”, because “it is unethical to confine and reproduce animals for species preservation” [46].

Zoo actors representing a justificatory discourse situated “animal rights” as idealistic. Chai (Ménagerie) denounces animal rights activists who advocate releasing un-adapted zoo animals into the wild, condemning them to die. This happened at the SGPZ in 2017, when, in response to animal rights campaigns, the zoo released two dolphins into the wild. Having lived 20 years in captivity, these individuals likely did not survive [47]. Jacques and Quertier (PZP) highlight that, “This is the whole paradox of working in a zoo. We know that ideally animals shouldn’t be in cages. In an ideal world, we don’t exist”. For them, a non-ideal world in an environmental crisis requires an organization dedicated towards reconnecting people to nature.

Displaying individual animals in captivity presents zoo actors with the first of two inherent dilemmas: how to truly consider wild animal interests within the constraints of the zoological park. Asking himself, “are anti-speciesists raging madmen, or does their discourse have a certain validity?”, Saint Jalme (Ménagerie) states that the past few years of modern zoo discussions on their environmental conservation mission and responsibilities towards individual animal welfare encouraged him to reflect on “the mosaic relationship of intelligence and consciousness in animals”. For Saint Jalme, knowledge of animal behavior that he had previously used for collectivist goals in conservation was now shedding light on the value of animal individuals. Referring to Nénette, an orangutan at the zoo, Saint Jalme expresses that, “an increasing awareness of animal consciousness led me to question myself regarding the animals we have in captivity and I admit, when we realize that Nénette has spent 50 years in captivity, it touches me somewhere, and, since I can’t do anything other than keep them here, somewhere I put on blinders. So, I will do everything to improve their quality of life”. Kim, B. (SGPZ) also emphasizes, “For these animals sacrificing themselves [for conservation], it is our responsibility to make sure they live with the best welfare possible”.

Zoo actors situated positive welfare as an ethical duty to adhere to captive animal interests, giving them opportunities to freely express their natural behavior and effectively represent their wild counterparts. Saint Jalme (Ménagerie) believes that, similarly to anti-zoo organizations, there exists “an individualization of animals at the zoo, like in [animal] rights which demands that animals be considered—that we give them individual rights”. Within the zoo, “we will pay attention to a certain individual and each individual in the group has his or her value”. For Bourgeois (Ménagerie), giving animals the freedom to choose and control changes to their environment is emblematic of the current welfare goals and practices of modern zoos: “Once all the basic needs are met, welfare is to provide the animal with choice and control over his environment. So, stop controlling him and make him in control”. Saint Jalme (Ménagerie) also echoes that “welfare means allowing the animal to master his or her environment”. Using animal welfare science to find possibilities for animals to express control over their own preferences, zoos hope to create “the best possible captive environments: one where animals would choose to stay even if they could leave” [48] (p. 241). Offering animals ways to express choice and control over their preferences has become central to animal welfare protocols of the EAZA and the AZA. Such choices include when and where to interact with conspecifics or individuals of other species, which foods to eat, whether to stay in an exterior or interior enclosure, or else to hide or remain in public view. Environments that provide control over such choices include spacious, varied exhibits, and training programs which individuals can opt in or out of. Bourgeois (Ménagerie) adds that it is vital for animals “to be able to choose their social systems. In nature, they are in fission-fusion—sometimes they like to be alone, sometimes they regroup to eat, at night they may separate. To reproduce this in captivity means having multifunctional enclosures, compartmentalized where animals can choose for themselves where they want to go”.

Both the PZP and Ménagerie have evolved to prioritize the welfare and autonomy of animal individuals in their practices and exhibit designs. The PZP, remodeled in 2014, converted most exhibits into multi-species exhibits, providing animals with opportunities to interact with members of other species. The zoo also expanded enclosures to be more spacious and varied with options for animals to hide form the public eye. Unlike the PZP, the historically classified Ménagerie has less flexibility with expanding or remodeling enclosures. As a result, in the past thirty years, it has compensated for lack of space by housing smaller animals and designing enclosures with more vertical space. As stated by Bourgeois (Ménagerie), “the Ménagerie is in perpetual evolution and there is a guideline to make bigger species leave, focus on small to medium sized species with an accent on conservation”. For example, red pandas and binturongs, both small-sized, critically endangered species, have replaced the large bears originally housed in the Ménagerie’s “fosse aux ours” (bear pit).

Within the past decade, the SGPZ has also made significant strides to improve animal welfare by expanding enclosures, providing positive reinforcement training, and adding enrichment to animals’ routines [9,49]. However, for Ma (Animal Happiness Lab), the zoo still has a long way to go: “We are not at the level of giving animals choice and control. The problem is that most Korean zoos don’t practice enrichment. Even at the Seoul Zoo, enrichment training is provided only two or three times a week instead of once a day, which is the standard”. Defining animal welfare as a “consistent effort” to expand animal happiness, she points out that Korean zoos have a shortage of staff with the qualifications and knowledge needed to ensure animal welfare best practices. However, the SGPZ’s recent membership to the AZA in 2019 has provided the organization with access to welfare resources and expert mentorship, something that “broke the mold” for Ma, in terms of providing zoo animals with positive welfare beyond basic physiological needs and mental stimulation. She hopes the SGPZ can maintain its accreditation status to continue to have access to these resources.

Addressing animal interests, however, reveals a second dilemma for zoo actors, who find themselves balancing these with the interests of their visitors. Bourgeois (Ménagerie) points out that giving animals choices in zoos comes with certain constraints. Although allowing an animal free access to enter and exit their enclosure at will is a priority, “Unfortunately, we cannot do this for all species. For example, we have security restrictions for the panthers. But, in the future, we’ll have to think about giving them permanent free access so that they have the choice to be in the space that they want”. Additionally, to move away from traditional entertainment, such as hand feeding or petting, that occurred up until 2013, the Seoul Zoo teaches about “zoo etiquette”, enrichment, and positive reinforcement training [9,49]. Similarly, the PZP informs visitors that animals require privacy and may not always be visible. After exhibits were remodeled in 2014, animals were offered larger enclosures with more hiding spots [41]. Prioritizing individual animal welfare in this way can conflict with what the public expects in terms of entertainment. At the Seoul Zoo, many visitors have trouble understanding why they can no longer feed or pet baby animals. Zookeepers expressed frustration when this interfered with their education mission [9]. According to Jacques and Quertier (PZP), when animals are barely visible because of their environment, visitors can sometimes feel as though they had wasted their visit: “That was destabilizing for visitors when we reopened the zoo. It was really something that people who had an image of the old zoo and the desire to see animals that they knew did not understand. Those that did not understand did not return”. As a response to frustrated visitors, educators emphasize the zoo as being the animal’s domain: “We are in the animal’s home, and not the other way around”.

### 4.2. Captive Breeding

As part of their justificatory discourse, modern zoos concern themselves with ex situ conservation, breeding individual animals in captivity to maintain species populations outside their natural environment and exchanging individuals between organizations. Taking up the “millennium ark” role, zoos justify captive breeding as a method of preserving stocks of animals available for in situ conservation in the distant future. Until then, to maintain genetically viable populations in the long term, captive breeding must ensure that these zoo populations maintain 90% of the original wild population’s genetic variation over 100 years [50,51]. This reflects the population management goals of global and regional zoo associations. Zoo members therefore actively participate in species management programs (SMP), established as a way of “managing a particular taxon with a globally agreed set of goals, while building upon and respecting existing regional processes” [52] (p. 1). These include guidelines set by the EAZA and the AZA, which facilitate collaboration between members through animal exchanges, conservation strategies, and welfare resources.

We found that actors representing an abolitionist discourse considered captive breeding as problematic because of its failure to integrate the intrinsic rights of individual animals—especially that of bodily integrity. This was informed by how actors framed individual animals as sentient persons and supported by their views of animal rights and conservation, which rejected ex situ conservation as a viable way of preserving biodiversity. In contrast, for actors representing a justificatory discourse, the main dilemma inherent in captive breeding was how to maintain genetically viable populations without significantly compromising the welfare of individual animals involved. We found that zoo actors framed individual zoo animals as reproductive components of genetic lineages. This role was reinforced by the situated meanings actors ascribed to the relationship between conservation and animal welfare, with the former perceived as the preservation of genetic patrimonies, and the latter as a means of maintaining individual animals functional in their reproductive roles.

Because actors representing an abolitionist discourse framed animals as sentient persons, they focused their concerns on individual animals’ loss of liberty and bodily integrity over species or ecosystem status. As Sanvisens (PAZ) points out, there are “Two things for us that are very important to distinguish: species and individuals. So, for species, the question is, will there be orangutans tomorrow? But what concerns us, are individuals. And, that’s where we seem to have trouble finding an understanding with zoos”. Referring to Nénette, the fifty-year-old orangutan at the Ménagerie, Sanvisens asks, “Does this person, this individual, does she suffer or not? That’s what’s going to interest us”. By calling Nénette a person, Sanvisens applies the abolitionist frame that individual living beings are persons with the basic moral rights to life, liberty, and bodily integrity [14].

Actors representing an abolitionist discourse situated “conservation” as in situ only, and condemned ex situ conservation as ineffective. PAZ presents conservation as a “false alibi” of zoos, stating that conservation politics should only concern itself with “protecting habitats” and the “fight against poaching”. In the declaration of animal rights co-authored by LFDA, Article 8 states that, “Any act compromising the survival of a wild species, and any decision leading to such an act constitutes genocide, meaning a crime against the species”, and that “the massacre of wild animals, pollution, and destruction of biotopes are genocides” [53] (p. 2). Bachelard (LFDA) and Morette (Code Animal) believe that, because wild animals belong in nature, conservation must be practiced in situ. Bachelard states, “In situ conservation, in our opinion, is the unique mode of conservation that should be put in place. This means that the right for any species not to disappear by humanity’s doing is currently not respected because we are going through a sixth mass extinction that is primarily due to human activities. So, we have somewhere a duty to make sure that this slows down, that this does not happen, or else intervene to improve the situation and make sure that species do not disappear. And when it comes to interventions, we believe zoos are completely ineffective and that in situ conservation can work if it is well managed”.

Because abolitionist actors framed individual animals as sentient persons with intrinsic rights and situated effective conservation as in situ only, they condemned captive breeding as a method intent on keeping a supply of captive animals within zoos for profit rather than for conservation in the wild. Gérôme (FBB) recognized that zoos can hold a significant role in in situ conservation projects—many of the NGOs her organization works with are themselves financially supported by zoological parks. However, she believes the conservation mission of zoos is mostly to have a good conscience and that their primary goals are lucrative: “What zoos claim is that they practice conservation. This means they keep endangered species from disappearing habitats, so they don’t disappear. Sure, why not? But we have existing sanctuaries and refuges that already do this, and not for business, since they are non-profit organizations”. Sanvisens (PAZ) views zoo animals as forced to be in a “state of permanent reproduction” to maintain the existence of the organizations. Arguing that animal transfers and forced reproduction places unnecessary stress on animals, Bekoff (University of Colorado) advocates for “no more captive breeding, no more shipping animals around as mating machines to make more animals who are going to be living in captivity”. To facilitate a transition into a world without zoos, Bachelard (LFDA), asks that these organizations “stop reproducing animals. Because, if zoos exist, it’s because there are reproduction programs put in place and zoos exchange animals to continue to sustain themselves. If we stop reproduction, after a time, there won’t be any animals left [in zoos]”.

However, zoo actors representing a justificatory discourse identified that the main dilemma inherent in captive breeding was maintaining the genetic integrity of populations, while also providing for the interests of individual animals through positive welfare. Zoo actors recognized that the production and displacement of reproductively functional individuals between institutions inevitably implies going against individual animal interests. Saint Jalme (Ménagerie) reflects that: “Every time we put in place an action that will isolate individuals, put contraception in place, or cull an animal, these are actions that will impact animal welfare and if we want to manage populations in captivity for the long term, then, yes, we find ourselves in conflict between welfare and conservation tradeoffs”. Similarly, Kim, B. (SGPZ) highlights that the most difficult decisions she has encountered involve figuring out how to properly manage species populations: “Unconditional breeding restrictions can lead to species reductions if they can’t reproduce. And if you send them to another zoo, then you need to make sure they have the right amount of habitat space. This is a real dilemma”. Bourgeois (Ménagerie) points out that translocating animal individuals, from one zoo to another, subjects animals to high amounts of stress and impositions detrimental to animal welfare: “For conservation purposes, we will be asked to, for example, stop the reproduction of an animal or we will be asked to translocate animals—such and such an animal must go to this place to reproduce with such and such individual. So, these are things that are imposed—animals can’t choose their partner, they can’t choose the moment when they are in contact with others. They’re raised with their parents and then one day we take them away without warning. We jump on them, put them in a truck, have them travel several kilometers and they arrive in a place they don’t know. So all this is extremely perturbing, and this is completely counterproductive to animal welfare”.

To justify captive breeding, zoo actors framed the role of individual animals in zoos as reproductive components of biotic wholes. Articles in journals printed by the Société d’Encouragement pour la Conservation des Animaux Sauvages (SECAS), a charity association partnering with MNHN zoos, refer to several “reproductive couples” of various species housed in zoos. One describes Nénette, the oldest orangutan at the Ménagerie, as, “an excellent mother sociable and gentle, [who] now lives a peaceful retirement”, after having contributed four offspring to the captive orangutan population in European zoos [54]. Monfort (SNZP/SCBI) refers to zoo animals as comprising “insurance populations against extinction”, or as “a demographic and genetic reservoir as a hedge against extinction”.

When justifying captive breeding practices for ex situ conservation, zoo actors, especially zoo managers, situated the meaning of “conservation” in zoos as preserving genetic patrimonies through managing populations ex situ. Joly (MNHN) reflects this mentality: “Well, the word “conserve” says a little about its meaning. It’s to preserve in time, from the erosions and the aggressions that can arise. So, for living populations, it’s a bit more complicated than dead objects. For dead objects, we know to put them in the right lighting, temperature, and humidity. For living populations, we conserve lineages. We are essentially conserving a genetic patrimony”. Saint Jalme (Ménagerie) adds that conservation means managing nature: “Since humanity is expanding, we have to manage nature with humans and reconcile people and nature”. For Monfort (SNZP/SCBI) and Pitt (SCBI), conservation also involves a heavy focus on species management and preserving the genetic integrity of animal populations for the future.

To fulfill conservation goals through captive breeding, actors representing a justificatory discourse situated “animal welfare” as a means of keeping animals functionally reproductive. Joly (Jardin des Plantes) recognizes that “Often when animals are not well, they don’t reproduce. They must be well to reproduce well. The connection could be there. Since for me, conservation is a lineage. It’s not the individual”. The role of welfare in conservation, therefore, serves to maintain the individual components of a larger system alive and comfortable at the most fundamental level, such that they are reproductively functional. Song (KNPS) reflects this perception of welfare as instrumental to conservation in his in situ fieldwork with Asiatic black bear conservation: “Restoring the species means getting the bears to reproduce and making sure they disperse. Beyond welfare, if this doesn’t work, then we cannot restore the bears. Caring for the bears’ welfare is part of [conserving them], but it is not more important”. In the world of ecological restoration, giving individual animals good welfare is useful if it enables reproduction, dispersion, and public support for conservation.

Actors representing a justificatory discourse, however, tended to situate conservation and animal welfare as fundamentally separate concepts when discussing captive breeding priorities. Smith (SNZP) believes, “Good welfare doesn’t necessarily have anything to do with conservation, and conservation doesn’t have anything to do with welfare”. She explains that one could successfully practice conservation without having to worry about mistreating an animal individual. Joly (Jardin des Plantes) also saw no concrete relationship between welfare and conservation: “Like this, spontaneously, I would say there is not necessarily a connection. That’s to say, you could conserve without being preoccupied by the way you conserve. Since, after all, this is about conserving a lineage. So, if animals are reproducing, that we have these lineages conserved, my goodness, we have achieved the conservation mission, since that is the objective. Honestly, when I think about it, I have never asked myself the question, but I don’t link the two notions. One can very well conserve independently, without worrying about animal welfare, as long as they reproduce”. As an animal welfare and research manager, Herrelko (SNZP), points out, “That’s a question that I haven’t really thought a lot about in terms of the definition of conservation, because I am so focused on welfare, and welfare does tie with conservation. [Conservation] is so broad and I so closely work in welfare that it’s never been something anybody’s ever asked me”. Yet, she underlines, “Everything we do in conservation, in terms of research has a welfare element. I’m hoping we can learn new ways to help beef that up, whether it’s pulling in the expertise of what we learn in animal management and bringing that to field sites”.

### 4.3. Culling

The culling or “humane killing” of healthy animals considered “surplus” is a potential consequence of captive breeding in zoological parks, especially if they wish to maintain the genetic integrity of their populations. Both the AZA and the EAZA recognize euthanasia as a method of managing populations of captive animals [55,56]. In 2014, the Copenhagen zoo euthanized a young healthy giraffe named Marius, because his genes were sufficiently represented within the giraffe population of the EAZA [24,57]. A 2020 article in *Le Monde* questioning the relevancy of zoos today states that, “Even if no other euthanasia has received as much attention since [Marius’ death], 3000 to 5000 animals continue to be killed every year in Europe for population management reasons in zoos” [58]. In response to public backlash from the Marius incident, the EAZA created a Communications Committee to provide transparency on “taboo subjects”, and “work on a communication strategy respectful of cultural differences in zoos whilst defending core EAZA values and principles” [59]. They also revised their statement on culling to reinforce their position on the practice, acknowledging that, “while EAZA members are ethically obliged to maximize the physical and psychological wellbeing of individual animals in their care, their responsibility for the fulfillment of defined conservation goals and the viability of the overall population may, under certain conditions, take precedence over the right to life of specific individual animals” [56] (p. 1).

For actors representing an abolitionist discourse, culling practices further reflected the failure of zoos to integrate the intrinsic rights of individual animals—namely the right to life. This perspective continued to be informed by how actors situated animal welfare as insufficient to provide for the interests of animals and in situ conservation as the only valid method of conservation. As a result, they attributed culling practices as an inevitable consequence of zoo breeding programs for lucrative aims. As stated by Bekoff et al., “zoos themselves create these moral conundrums”, because “using animals as breeding machines is good for business or, they claim, essential for conservation” [60] (pp. 46–47). Bachelard (LFDA) states, “For us, this is one of the reasons that zoos have no purpose. They claim to conserve species, so that’s why they make animals reproduce. But, when there are animals they don’t know where to place because there will undeniably and inevitably be problems with their genetics, they find themselves required to kill captive ‘surplus’ animals”. Gérôme (FBB) upholds that, “from the moment that you have a business, that you make money with a wild animal, even if many zoos claim or promote their willingness towards species conservation, this is still a business”. From this, she argues that, automatically, the business ventures of zoos cause animal exchanges for reproduction which result in healthy “surplus” animals for euthanasia.

To inform culling practices, zoo actors continued to frame individuals as reproductive components and situate the meaning of conservation as the need to maintain genetic lineages. To justify this practice, they leaned most on situating the meaning of welfare as “what the animal feels” and “the ability to express natural behaviors.” However, this resulted in four central dilemmas, inherent to culling practices for zoo actors.

First, although all interviewees working in zoos highlighted that the culling of healthy “surplus” animals was socially and culturally unacceptable in their respective countries, many did not view culling as a tradeoff between animal welfare and conservation priorities. With reference to culling practices, zoo actors representing a justificatory discourse situated the meaning of welfare as “what an animal feels”. This welfare position assumes most animals are not self-aware and that the experience of a painless “good death” neither enhances nor diminishes that animal’s welfare. EAZA therefore notes that “modern welfare science regards lack of life as a neutral position” [56] (p. 1). For Smith (SNZP) and Bourgeois (Ménagerie), the culling of “surplus” animals therefore does not present a welfare concern, but an ethical one. Romain (Akongo) asks: “From an ethical stance, do we accept to euthanize healthy animals under our responsibility? I think this situation doesn’t have a clear and simple answer. It’s really a societal issue at the ethical level on what is considered acceptable or not”. According to Saint Jalme (Ménagerie), “The public today is not willing to accept killing an animal for management reasons”.

Second, zoo actors themselves struggled with the ethics of euthanizing a healthy animal to optimize population management for conservation. Saint Jalme (Ménagerie) exclaims, “What do I think of this? It’s the politics of compromise and it’s case by case. But I’m conflicted by EAZA recommendations, which are ‘breed and cull’”. Although trained as population biologist, Saint Jalme finds himself at odds with the consideration of individuals and species conservation priorities: “This individualist vision of animals, I have it more and more despite having started working on population issues, reintroduction issues, and population reinforcement. [Back then], what was important was to succeed in nature, to reconstitute the species despite collateral damage. So, if we had to sacrifice an individual to produce three others, I had no qualms”. Duby (Ménagerie), reflects that, “Nobody suffers, except by dying. That’s the dilemma, the central question. Population management is very logical but taking away the life of a healthy animal—I have trouble accepting that. It’s very personal”. Another French veterinarian believes the sacrifice of individuals for the collective aims of conservation serves no concrete purpose: “Conservation is politics, so in the end it’s hollow. Welfare, on the other hand, is real. When I have a sick animal, I could care less if he’s part of some population for conservation, all I want is for him to be well, even if it implies euthanasia”.

Third, zoo actors found themselves having to decide between the welfare of individuals or the welfare of the group. In this case, actors situated animal welfare as “the ability to express natural behaviors”. Romain (Akongo) comments that alternative practices to culling, such as isolation or contraception, can, “risk causing a loss of natural behaviors related to reproduction. Do we privilege this behavioral diversity and think in terms of viable populations rather than at the individual level? The choices are complex”. Simon (RZHT) justifies the culling of individual dholes, a species of Asian wild dog, at his zoo, as necessary for the overall welfare of the population. Simon explains the importance of maintaining juveniles in the group, allowing them to express their natural reproductive behaviors, but resulting in an excess of “surplus” individuals: “if we were to suspend, even temporarily, reproduction in those groups, then there would be a carnage, I am convinced of that”. To recreate natural conditions, the EAZA recommends removing individuals at the adult or sub-adult stage, to mimic the dispersal and high mortality that naturally occurs at this stage in the wild. Bourgeois (Ménagerie) explains that, “this favors the group dynamic to allow mothers to continue to raise their young, because sometimes that is something that can be lost in a group and, for animal welfare, social enrichment is very important”.

Finally, culling practices involved zoo actors choosing between euthanizing a healthy animal or the potential for that animal to live a life of suffering. In the case of removing a “surplus” animal, sending the individual to a non-EAZA accredited facility, as an alternative, risks placing the individual in questionable welfare conditions for life. Saint Jalme (Ménagerie) describes the internal strife one faces as a manager when making these difficult decisions: “Well, these are part of things that are complicated to manage. Because you are at odds with your values, you are at odds with so many things. So, it’s more than a compromise. Here, you’re in a crisis”. Simon emphasizes that “We can’t allow ourselves to have animals in deplorable situations because we are unable to place them elsewhere”. In 2017, the Seoul Zoo encountered this type of dilemma when they re-homed Taiji, a dolphin who found himself alone in his enclosure after the decision was made to reintroduce the remaining two dolphins in the wild. As a Japanese sub-species, experts were concerned that reintroducing Taiji would disrupt the existing Korean pod of dolphins. Understanding that Taiji needed to be with conspecifics, the zoo decided to transfer him to a sub-par aquarium facility on Jeju island [61]. However, both Simon and Saint Jalme point out that, in similar situations, members of associations, such as EAZA, may choose to euthanize an animal over transferring him or her to an outside institution with inadequate facilities.

## 5. Discussion and Conclusions

This paper aimed to determine the extent to which, and how, zoological parks recognize the intrinsic value of wild individuals beyond their status as members of collective species or ecosystems. To do so, we analyzed two discourses on the relationships between animal rights, welfare, and conservation in zoos. Actors representing the first abolitionist discourse framed individual animals as sentient persons; whereas, those representing the second, justificatory discourse, framed them as “species ambassadors” and “reproductive components”. The analysis showed how the situated the meanings of conservation, animal welfare, and animal rights informed and supported the different perspectives on dilemmas inherent in zoo practices related to ex situ conservation.

First, actors representing an abolitionist discourse condemned zoos for violating the intrinsic rights of individual animals by breeding them in captivity for human enjoyment. These interviewees situated animal welfare as too subjective to fully integrate the needs and interests of “wild” individuals without liberty. This is consistent with the argument that the concept of “’unnecessary suffering’ is insufficiently precise and always makes animal well-being subservient to human interests” [62] (p. 166). Under an animal welfare ethic, individual animals can be sacrificed for the greater good, whereas the same would not be permissible for humans. In this case, the validity of individual animal suffering also changes according to what is necessary for human interests. For this reason, the ethic of animal welfare is often at odds with the animal rights ethic, which reflects Regan’s (1983) position rejecting species boundaries and advocating moral equality to life, liberty, and bodily integrity [14].

Second, actors representing a justificatory discourse framed zoo animals as instrumentally valuable “ambassadors” and “reproductive components” of larger collectives. This reflects a collectivist approach to nature, which “prioritizes the group over its individual constituents” and is dominant in conservation biology [6] (p. 1262). Despite “biodiversity” including both individuals and collectives, “conservation efforts have focused on the preservation of collectives, with wildlife individuals viewed and valued as instances of their type rather than unique and distinct organisms” [6] (p. 1262). Stibbe (2012) warns that the “discourse conducted at the level of mass and collective nouns has the potential side effect of distracting attention away from the direct relationships with individual animals: an individual can be seen, heard, and empathized with, but a ‘species’ cannot” [63] (p. 72).

Third, to justify captive breeding practices, actors representing a justificatory discourse viewed a relationship between animal welfare and conservation in terms of fitness, adhering to the biological function orientation of welfare, where “an animal has good welfare when, among other attributes, [he/she] grows well, is in good health, reproduces successfully, and is relatively stress free” [64] (p. 14). Used to assess “hindrances to achieving biological fitness, resilience, and performance”, this represents the bare minimum of zoos’ responsibility towards individuals [13] (p. 21). Yet, Beausoleil et al. point out that the tendency to base welfare solely on the physical fitness of individual animals in conservation biology goes against the most up-to-date animal welfare scientific studies, which “emphasize the dynamic integration of “fitness” and “feelings” (mental experiences) to holistically understand animals’ welfare states” [65] (p. 1). Solely considering the biological function orientation of animal welfare in fulfilling conservation outcomes reduces the intrinsic value of individual animals to expendable parts of a larger whole, and justifies zoo actions that encourage this. In these cases, only those welfare aspects relevant to aiding an individual in successfully reproducing with another were deemed relevant for conservation outcomes. According to the collectivist priorities dominating zoo conservation biology, those individuals who are no longer relevant, such as “surplus” animals who do not meet zoo breeding and exhibition requirements, can either be eliminated or prevented from reproducing [66].

Fourthly, although actors representing a justificatory discourse do not portray zoo animals as persons with the right to liberty, the evolution of welfare practices in AZA and EAZA institutions demonstrate that they value their autonomy and interests. Zoos argue that individual animals can flourish in captivity if they are provided with opportunities to practice choice and control over their captive environment. Allard and Bashaw (2018) describe this as a form of empowerment, where an animal can choose to react to and control changes to his or her environment [48]. Choice, defined as “the power or the liberty to choose between alternatives” signifies providing available options for individuals to express their natural behaviors [66] (p. 61). Practices that offer animals ways to express choice and control over their preferences represents the highest level of Maslow’s hierarchy of needs [17] (p. 8). These options allow zoo animals perceive themselves to be in control despite captivity [48]. An individual animal’s sense of control, or agency, is reinforced by his or her ability to have choices, and undermining this agency is harmful to his or her well-being [67]. This recognition of individual animal agency has become central to animal welfare protocols.

In conclusion, this analysis revealed two significant findings. First, actors representing the justificatory discourse of zoos failed to frame zoo animals as intrinsically valuable individuals with respect to ex situ conservation priorities. Second, within the constraints of the zoo, the intrinsic value of individual animals was, however, recognized through welfare practices and educational priorities focused on fulfilling animal interests and promoting an ethic of care.

First, because the nature of their organizations requires managing the lives of individual animals from birth until death in captivity, zoos do not fully integrate the intrinsic rights of animal individuals. For actors representing an abolitionist discourse, the moral dilemmas inherent in zoo practices could easily be avoided by abolishing captivity. Many of the ethical challenges between welfare and conservation faced by zoo personnel related to captive breeding and culling involved attempts to recreate “natural” conditions in artificial environments. In addition, to justify ex situ conservation practices, actors representing a justificatory discourse consistently referred to animals as members of collectives—from comprising genetic lineages to representing species. In these practices, welfare therefore became instrumental—a means of ensuring the functionality of these components and improving their effectiveness as ambassadors for their wild counterparts. Moreover, although zoo actors viewed death as neutral with respect to animal welfare, the ethical dilemmas that are inherent in the culling of healthy animals consisted of choices between whose welfare deserves the most consideration, suggesting that this practice also embodies tradeoffs between animal welfare and conservation priorities.

Second, although the justificatory discourse and frames make it impossible to see animals as intrinsically valuable individuals in practices such as captive breeding and culling, positive welfare practices, involving enrichment, choice, and control, highlight the intrinsic value of individual animals. Zoo educators also emphasize teaching visitors about respecting animals in their “domain”. Finally, current welfare practices, allowing animals to express their interests and autonomy in an environment that restricts their freedom, suggest that individuals do matter in zoos. The evolution of welfare practices in zoos indicate an emerging animal rights ethic based on interests, that works to bring forward the individual, while maintaining the zoological park as an organization considered necessary for conservation in a non-ideal world.

Overall, understanding the extent to which, and how, zoos recognize the intrinsic value of wild individuals can aid these organizations in developing conservation and welfare priorities that align with compassionate conservation principles and avoid intentionally harming animals. Though captivity may be deemed by some as deliberately harmful towards individuals, welfare practices that prioritize providing autonomy to zoo animals demonstrate that individuals do matter in the zoological park. In addition, understanding how zoo individuals matter has the potential to open a dialogue between opposing perspectives on the role of zoos and their duty of care with respect to addressing individual animal interests. Gray (2018) argues that compassionate conservation can help resolve and identify “areas of agreement and areas of dispute” between conservation and welfare priorities [68] (p. 1). Practically applying these principles could help zoos identify and resolve their own ethical dilemmas related to breeding captive wild animals in artificial environments, such as the welfare implications of managing “surplus” individuals.

## Figures and Tables

**Table 1:** Thirty-one interviews conducted in France from May to August 2019 and from March to October 2020.

Organization	Interviewee	Position
Ménagerie ^1^	Bourgeois, Aude	Veterinarian
	Chai, Norin	Adjunct Director/Chief Veterinarian
	Duby, Dylan	Veterinarian
	Hano, Christelle	Head Zookeeper
	Rey, Élodie	Curator
	Kayser, Pauline	Zookeeper
	Saint Jalme, Michel	Director
Parc Zoologique de Paris (PZP) ^1^	Jacques, Patricia	Educator
	Marquis, Olivier	Curator
	Morino, Luca	Curator
	Quertier, Élisabeth	Educator
Réserve Zoologique de la Haute Touche (RZHT) ^1^	Locatelli, Yann	Adjunct Director
	Simon, Roland	Director
Association Française des Parcs Zoologiques (AFdPZ) ^1^	Erny, Cécile	Director
La Fondation Droit Animal Éthique et Sciences (LFDA) ^2^	Bachelard, Nikita	Public Relations Officer
Animal Rebellion ^2^	“Boonkin”	Activist
La Fondation Brigitte Bardot (FBB) ^2^	Gérôme Delgado, Élodie	Adjunct Director, Animal Protection Division
Code Animal ^2^	Morette, Alexandra	President
Paris Animaux Zoopolis (PAZ) ^2^	Sanvisens, Amandine	President
Akongo ^3^	Romain, Amélie	Head Animal Welfare Specialist
Université Paris Créteil ^4^	Estebanez, Jean	Geographer
Liège Université ^4^	Servais, Véronique	Anthropologist
Muséum National D’Histoire Naturelle (MNHN) ^5^	Abourachid, Anick	Evolutionary Biologist
	Duboscq, Julie	Ethologist
	Joly, Éric	Director, Zoological and Botanical Gardens
	Maille, Audrey	Ethologist
	Mihoub, Jean-Baptiste	Ecologist/Conservation Biologist
	Petit, Odile	Ethologist
	Pouyedebat, Emmanuelle	Ethologist
	Sarrazin, François	Ecologist/Conservation Biologist
	Sueur, Cédric	Ethologist

^1^—Zoological parks and zoo associations; ^2^—animal rights organization; ^3^—animal welfare organization; ^4^—academic institution; ^5^—research organization.

**Table 2:** Thirty-four interviews conducted in the Republic of Korea from June to August 2017 and from April to November 2021.

Organization	Interviewee	Position
	Bae, Ju-Hee	Zookeeper, Species Conservation Education Center
Seoul Grand Park Zoo (SGPZ) ^1^	Choi, Jin	Curator
	Eo, Gyeong-Yeon	Coordinator, Research Laboratory
	Jeong, Yu-Jeong	Zookeeper, Species Conservation Education Center
	Kim, Bo-suk	Acting Director
	Kim, Min-Su	Action Officer, Conservation and Health Center Action
	Park, Seon-Deok	Team Leader
	Seon, Ju-Dong	Zookeeper
	Yeo, Yong-Gu	Director, Conservation and Health Center
	Yeom, In-Yeong	Education Coordinator
Korean Animal Rights Advocates (KARA) ^2^	Jeon, Jin-Kyeong	Executive Director
Action for Animals ^4^	Jeon, Chae-Eun	Representative
Animal Welfare Awareness Research, and Education (AWARE) ^4^	Lee, Hyeong-Ju	Representative
Animal Happiness Laboratory ^4^	Ma, Seung-Ae	Veterinarian, Representative
Ewha Women’s University ^3^	Choi, Jae-Cheon	Chair Professor, EcoScience Division
	Name Withheld	Researcher
Mokpo National University Institution for Marine and Island Cultures ^3^	Hong, Seon-Ki	Director, Center for Island Sustainability
	Kim, Jae-Eun	Library Studies Researcher
Jeju National University ^3^	Kim, Byeong-Yeop	Professor, Fisheries
National Institute of Ecology (NIE) ^5^	Kim, Yeong-Jun	Director, Animal Care Laboratory
	Jang, Ji-Deok	Department Head, Animal Care Laboratory
	Jeong, Gil-Sang	Researcher
	Ryu, Heung-Jin	Researcher
	Woo, Dong-Geol	Researcher
Marine Biodiversity Institute of Korea (MABIK) ^5^	Ahn, Yong-Rak	Department Head, Classification laboratory
	Han, Dong-Wook	Director
Korean National Park Service (KNPS) ^5^	Jeong, Dong-Hyeok	Director, Wildlife Medical Center
	Kim, Eui-Kyeong	Conservation Biologist, Mammals
	Kim, Jeong-Jin	Technical Team Leader, Species Restoration Technology Department
	Kwon, Yeong-Su	Conservation Biologist, Birds
	Name Withheld	Veterinarian
	Name Withheld	Researcher
	Song, Dong-Ju	Director, Jirisan Asiatic Blackbear Restoration Program
	Song, Jae-Yeong	Conservation Biologist, Reptiles and Amphibians

^1^—Zoological parks and zoo associations; ^2^—animal rights organization; ^3^—animal welfare organization; ^4^—academic institution; ^5^—conservation research organization.

**Table 3:** Fourteen interviews conducted in the United States of America from March to December 2020.

Organization	Interviewee	Position
Smithsonian National Zoological Park (SNZP) ^1^	Bernardoni, Elise	Assistant Director, Education Programs
	Herrelko, Betsy	Assistant Curator, Animal Welfare and Research
	Hill, Kristin	Supervisor, Conservation Engagement
	Monfort, Steven	Director, SNZP and SCBI
	Smith, Brandie	Associate Director, Animal Care
	Name Withheld	Educator
Smithsonian Conservation Biology Institute (SCBI) ^2^	Comizzoli, Pierre	Chair, Research, Animal Care and Use Committee
	Leimgruber, Peter	Head, Conservation Ecology Center
	Mcshea, William	Wildlife Ecologist
	Name Withheld	Representative
	Pitt, Will	Deputy Director
George Mason University ^3^	Name Withheld	Professor, Conservation Biology
University of Colorado Boulder ^3^	Bekoff, Marc	Ethologist
World Conservation Society (WCS) ^4^	Robinson, John	President

^1^—Zoological parks and zoo associations; ^2^—research organization; ^3^—academic institution; ^4^—conservation research organization.

## Data Availability

Restrictions apply to the availability of these data. Data in the form of recorded interviews are not publicly available to protect the privacy and confidentiality of research participants. Other data acquired from public documents are available in the references section of this article.
